# Rescuing biogeographic legacy data: The "Thor" Expedition, a historical oceanographic expedition to the Mediterranean Sea

**DOI:** 10.3897/BDJ.4.e11054

**Published:** 2016-12-22

**Authors:** Dimitra Mavraki, Lucia Fanini, Marilena Tsompanou, Vasilis Gerovasileiou, Stamatina Nikolopoulou, Eva Chatzinikolaou, Wanda Plaitis, Sarah Faulwetter

**Affiliations:** 1 Institute of Marine Biology, Biotechnology and Aquaculture, Hellenic Centre for Marine Research, Heraklion, Crete, Greece; 2 Australian Museum Research Institute, Sydney, NSW, Australia; 3 Institute of Oceanography, Hellenic Centre for Marine Research, Anavyssos, Athens, Greece; 4 University of Patras, Department of Biology, Laboratory of Zoology, Rio, Patras, Greece

**Keywords:** Marine biodiversity, Rhodophyta, Polychaeta, Clupeiformes, Historical dataset, Danish Oceanographical Expedition, Digitization, Data management, Data rescue, Data archaeology

## Abstract

**Background:**

This article describes the digitization of a series of historical datasets based οn the reports of the 1908–1910 Danish Oceanographical Expeditions to the Mediterranean and adjacent seas. All station and sampling metadata as well as biodiversity data regarding calcareous rhodophytes, pelagic polychaetes, and fish (families Engraulidae and Clupeidae) obtained during these expeditions were digitized within the activities of the LifeWatchGreece Research Ιnfrastructure project and presented in the present paper. The aim was to safeguard public data availability by using an open access infrastructure, and to prevent potential loss of valuable historical data on the Mediterranean marine biodiversity.

**New information:**

The datasets digitized here cover 2,043 samples taken at 567 stations during a time period from 1904 to 1930 in the Mediterranean and adjacent seas. The samples resulted in 1,588 occurrence records of pelagic polychaetes, fish (Clupeiformes) and calcareous algae (Rhodophyta). In addition, basic environmental data (e.g. sea surface temperature, salinity) as well as meterological conditions are included for most sampling events. In addition to the description of the digitized datasets, a detailed description of the problems encountered during the digitization of this historical dataset and a discussion on the value of such data are provided.

## Introduction

Historical data represent an invaluable source of information and are of paramount importance in establishing baselines for present and future studies, especially when dealing with global change. Marine meteorological historical datasets offer past climate information collected from early historical ship records that can help to create a more complete picture for global climate change research and help to establish effective predictive models and references for instrument calibration (Folland and Parker 1995, Woodruff et al. 2005, Brunet and Jones 2011). Historical biodiversity data, i.e. information on the past distribution of species, can help detecting the effects of climate change on range shifts, the distribution of invasive species, and the loss of habitats and species (Graham et al. 2004, Shaffer et al. 1998, Tingley and Beissinger 2009). The digitization of such type of data is a challenge in terms of identification and selection of literature, as well as human effort, but it returns information of an enormous value. The present study describes four datasets which were digitized, quality controlled and integrated into a harmonized dataset from publications of the Danish Expeditions to the Mediterranean Sea and adjacent marine areas during 1908-1910 and re-publishes: (a) the list of samples collected (including location and sampling method details), (b) the related hydrographical and environmental parameters and meteorological observations, and (c) the spatio-temporal occurrences of selected taxa (i.e. calcareous rhodophytes, pelagic polychaetes, and the fish families Engraulidae and Clupeidae).

**Figure 1.**
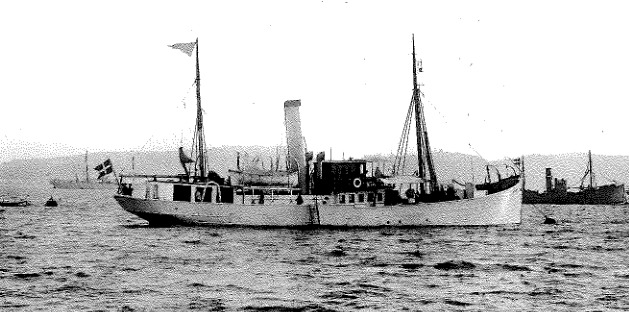
The Danish Research Steamer "Thor". Image from Schmidt (1912).

The core of the integrated datasets comprises the Expeditions of the Danish research vessel “Thor” (Fig. 1), which took place in the Mediterranean during the winter of 1908–1909 and in the summer of 1910. These expeditions were not independent cruises but had been planned as a continuation of several Danish oceanographic research expeditions in the Atlantic Ocean since 1903. During these expeditions, equal effort was dedicated to collecting both biological and biogeochemical data in order to be able to relate species occurrences to environmental conditions. Sampling stations were selected explicitly in order to compare the species composition between areas with different biogeochemical characteristics. Towards this end, although the two expeditions focused mainly on the Mediterranean Sea, some stations in the adjacent areas of the Atlantic Ocean, as well as in the Sea of Marmara and the Black Sea, were also surveyed.

The "Thor" Expeditions fell into a period of general interest in the exploration of the oceans, and may be listed among other famous expeditions such as the global Challenger Expedition, the Siboga expedition in Indonesia, or the Pola expedition in the Mediterranean Sea (Schefbeck 1996), to name just a few. The Danish Oceanographic Expeditions (on various research vessels) in the first 30 years of the 20^th^ century were facilitated by the leadership and enthusiasm of Johannes Schmidt, who also succeeded in procuring the funds for these expeditions. The Thor expeditions were exceptionally well planned and documented, and the research questions investigated were not simply of an exploratory or observational nature. In contrast to many expeditions of the late 19th century, the aim of this survey was not simply the discovery of rare or new taxa; instead, it focused on: (a) obtaining a thorough understanding of the hydrography of the Mediterranean Sea, the Atlantic Ocean and their merging zone, (b) obtaining information on the most common organisms, studying their occurrences (and absences) in relation to environmental factors at different depth layers, and (c) gaining a better understanding of the life cycle and life history of the studied species. Most of the sampling sites were open sea stations, while coastal areas were only sampled occasionally and not systematically. Additional samples were collected by other vessels (commercial and scientific) after the completion of the two main expeditions of "Thor" to help individual researchers to fill the gaps in their data.

The results of these expeditions were presented in three multi-part volumes published from 1912 to 1939 (Table 1). Apart from a series of hydrographic studies which were published mainly in the introductory volume (Volume 1), as well as in Volume 3, the biological data were published independently per taxonomic group in Volume 2. These publications were heterogeneous regarding their format and content; some focused on the life history of a single species, while others provided taxonomic reports of larger groups (e.g. Amphipoda) and stretched over several papers. Depending on the specific research under investigation, the authors used information not only from the Mediterranean "Thor" expedition, but to a varying extent also from the preceding Atlantic expeditions, as well as from the Danish research expeditions aboard the vessels “Pangan” (1911) and “Dana” (1921 and 1930).

This paper describes the process of digitizing the biogeographic data and sampling metadata of four publications included in Table 1: (i) Schmidt (1912), containing information on the expeditions, sampling stations, coordinates and relevant metadata (e.g. abiotic measurements); (ii) Lemoine (1915) on the taxonomy and distribution of calcareous rhodophytes; (iii) Wesenberg-Lund (1939) on the taxonomy and distribution of pelagic polychaetes; and (iv) Fage (1920), describing the development of the larval stages and the distribution of species in the fish families Engraulidae and Clupeidae. The specific four publications were selected in order to cover different habitats and organisms: from algae to animals, from pelagic to rocky shore species, and from larvae to adults. In addition, the team had sufficient taxonomic expertise on these groups to deal with problems arising during the quality control of taxonomic information.

## Project description

### Study area description

The 1908–1910 "Thor" expedition focused on the Mediterranean Sea and the adjacent waters of the Atlantic Ocean, the Sea of Marmara and the Black Sea; additional stations from the Atlantic are included in the digitized datasets since they were reported in the individual biological publications.

### Design description

During the two core expeditions of 1908–09 and 1910, 1,102 samples from 261 stations were collected. However, as the publications aimed at providing a more comprehensive picture of hydrographic and biological patterns across different geographic areas, they also included selected data from previous "Thor" expeditions in the North Sea and the North-Eastern Atlantic (1905–1906) and from other commercial and research vessels in the Mediterranean (1911–1912). These publications are also included in Schmidt (1912) and thus the introductory volume includes 1,566 samples from 443 stations.

**Figure 2.**
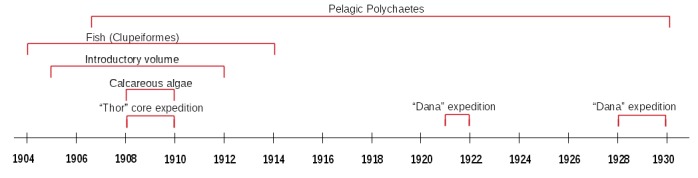
Timeline showing the different expeditions and the temporal coverage of the digitized publications.

**Figure 3.**
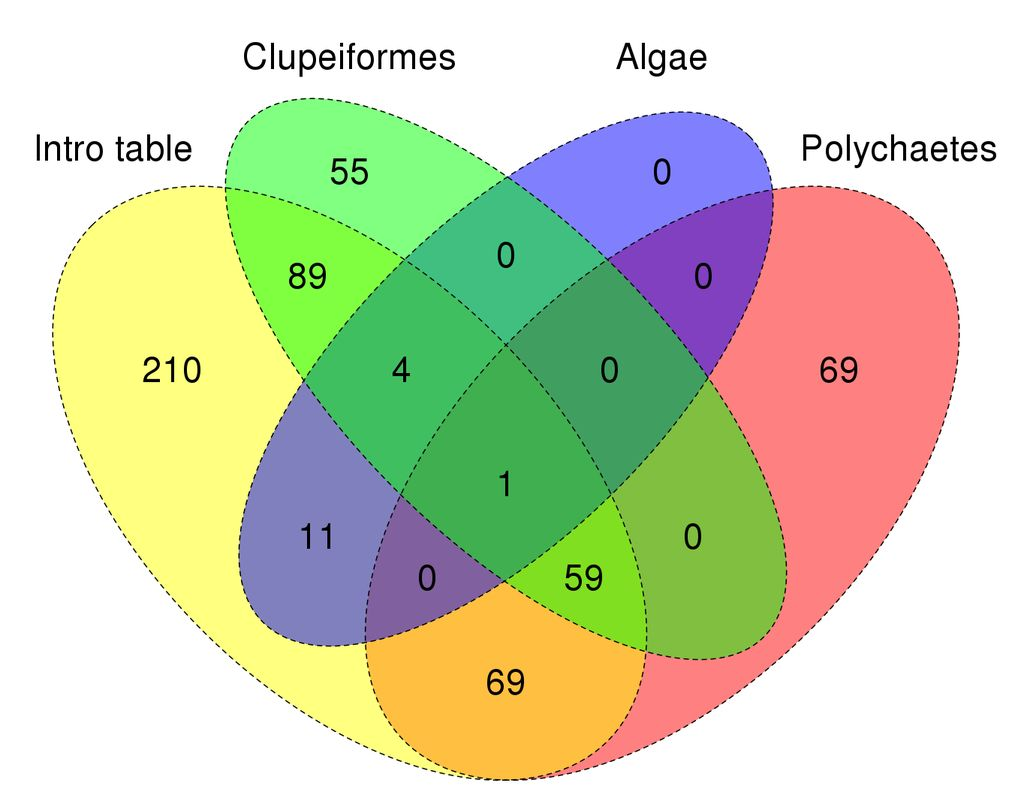
Venn diagram showing the overlap of sampling stations across the four digitized datasets.

The individual biological publications did not strictly analyse only the data from these stations but combine samples from various oceanographic expeditions from 1904 to 1930, including those of the research vessel “Dana” from its global campaign in the years 1921 and 1930 (Fig. 2). Thus, the combined datasets digitized and re-published herein finally comprise information from a total of 567 stations and 2,043 samples, which partially overlap (Fig. 3).

***Overview of the four digitized datasets***

*Introductory table (sampling and environmental information)*

The introduction to the Danish Oceanographical Expedition by Schmidt (1912) includes a series of tables containing information on all samples collected during the core expedition of the "Thor" to the Mediterranean in 1908-1909 and 1910 (pages 25–41), additional tables listing samples on the related expedition of the "Thor" in the Atlantic Ocean in 1905 and 1906 (pages 41–47) and supplementary samples taken in 1911 and 1912 by other vessels in several locations in the Mediterranean Sea (pages 47–49). Additionally, the introduction contains information on the characteristics of the research vessel, details on the sampling gears and a description of sampling methods. The main table describes all samples and lists relevant metadata such as station number and position, depths, sampling date and time, bottom characteristics, gear used (including the length of wire for nets and the duration of the haul) and environmental parameters (see also below, section "Sampling methods").

*Pelagic polychaetes* (Wesenberg-Lund 1939)

This publication describes the pelagic polychaetes (excluding Tomopteridae), collected from various cruises of the "Thor" and additional vessels (1905–1911), as well as samples from the "Dana" expeditions (1921 and 1930), including a few samples from the tropical Eastern Atlantic. The paper focuses on taxonomic treatments, including descriptions and illustrations for each species, but provides full details on the samples each species was found in.

Fish (Clupeiformes) (Fage 1920)

This publication includes data from various "Thor" expeditions from 1905–1910, but also includes information from other vessels, encompassing in total a period from 1904–1912 and a geographic area including the Atlantic, the Mediterranean and the Suez Canal. The paper focuses on the life cycle and development of commercially important species of the familes Engraulidae and Clupeidae (e.g. sardines, sprats, anchovies) and analyses only larval stages. Abundances per larval size class are reported for each station, aiming at assessing seasonal fluctuations and productivity of different regions in the study area. Samples, their metadata, and abundances per size class are reported in tabular format for each species.

In addition to the data collected by the research vessels, Fage (1920) also presents a time-series of twelve years of fish captures (“sardines”) by the fishermen of Colliure (southern France), showing defined seasonality and allowing for a comparison, within the same report, between basic data related to larval growth and data related to commercial fishing. The digitization of these "fishermen data" was considered also of historical value and is included here as Suppl. material 5.

*Calcareous algae* (Lemoine 1915)

This dataset includes taxonomic descriptions, drawings, global distribution maps and other remarks for the calcareous algae collected in the framework of the expedition. The paper focuses on taxonomic treatments, including descriptions and illustrations of species and listing the date and location it was found, but omitting additional information on coordinates, sampling dates, depth or gear. These metadata could in some cases be inferred from the introductory table, but in most cases this proved impossible due to lack of details given.

**Figure 4.**
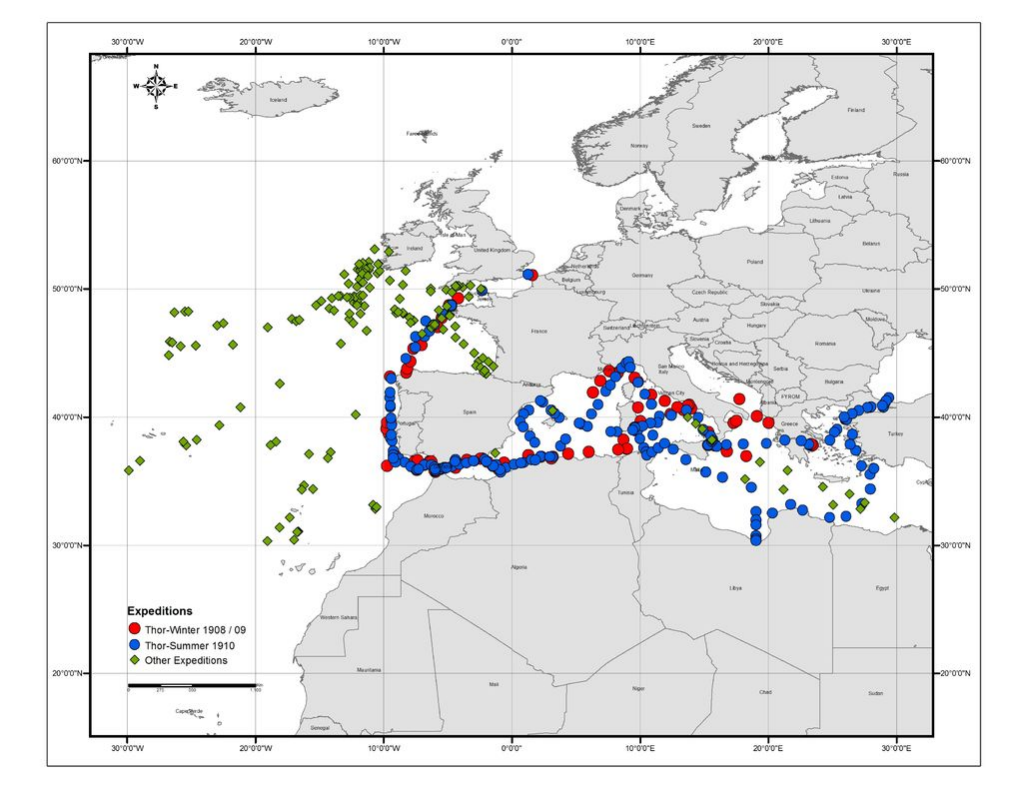
Map of the stations sampled by Thor and other vessels during the core expeditions of 1908–1909 and 1910 and additional expeditions from 1905–1906 and 1911–1912 (stations listed in the introductory table in Schmidt 1912).

**Figure 5.**
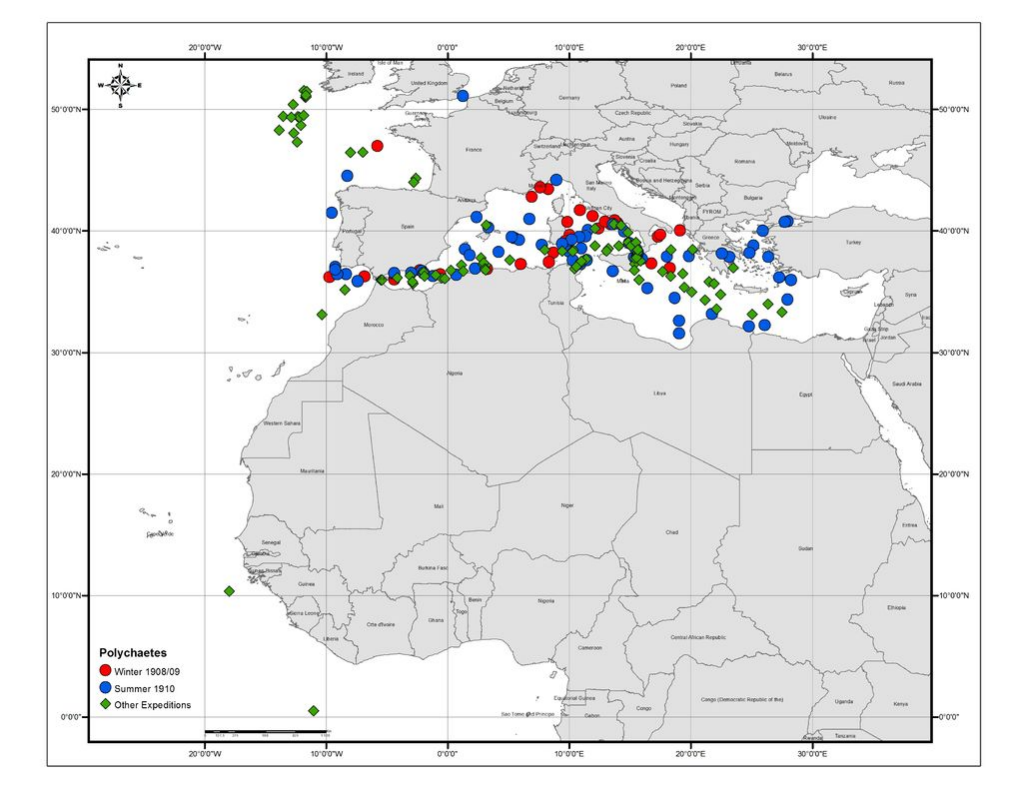
Map of sampling stations where pelagic polychaetes were collected.

**Figure 6.**
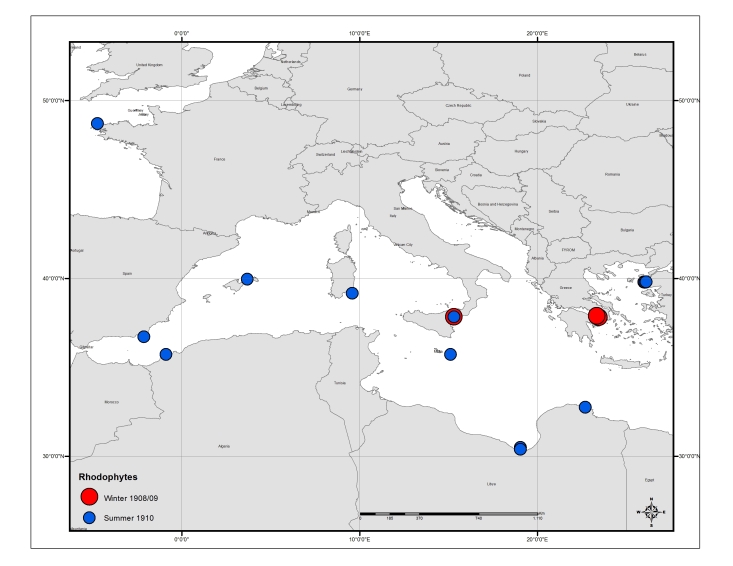
Map of sampling stations where fish (Clupeiformes) were collected.

**Figure 7.**
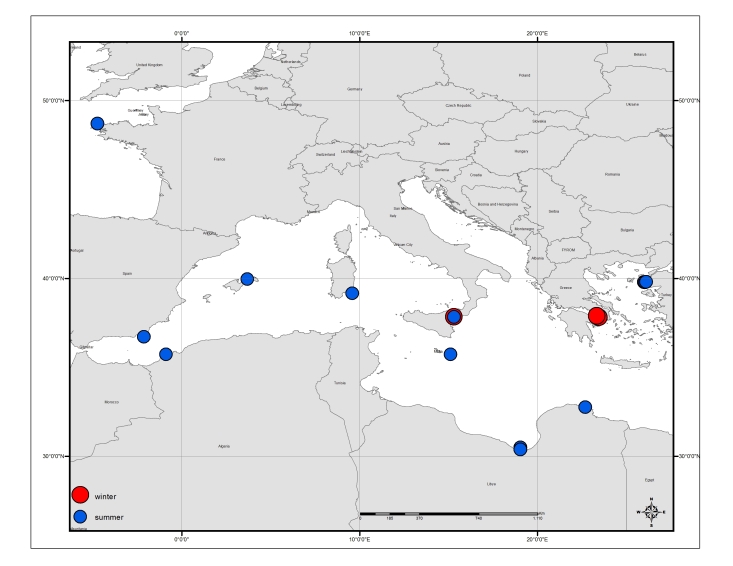
Map of sampling stations where calcareous algae (Rhodophyta) were collected.

Table 2 provides an overview of the characteristics and coverage of the four individual datasets; Figs 4, 5, 6, 7 show the sampling stations of each of the datasets. The datasets can also be viewed through the MedOBIS data viewer (account required) and downloaded in various formats.

### Funding

This work was supported by the LifeWatchGreece Research Infrastructure (MIS 384676), funded by the Greek Government under the General Secretariat of Research and Technology (GSRT), ESFRI Projects, National Strategic Reference Framework (NSRF). The original "Thor" expedition was funded by the Carlsberg Foundation (Copenhagen).

## Sampling methods

### Sampling description

The research steamer “Thor” was 35 m long and 6.5 m wide and was equipped with laboratory facilities for oceanographic surveys. The main types of sampling gear used during the “Thor” expedition and specifically for the four examined datasets are described below and listed in Table 3. In addition, film material recorded during the Danish Oceanographic Expedition Around The World (1928-1930) on the research vessel “Dana” documents the equipment of the ship and the use of the gear, much of which was used in a similar fashion during the "Thor" expeditions. Schmidt (1912) provides an extensive description of different types of sampling gear and their use on board.

For horizontal bottom and pelagic trawling, two models of young fish trawls ("Petersen's young fish trawl") were used, one 7 m long net with an opening of 200 cm diameter (Y 200), the other 8.5 m long with an opening of 330 cm (Y 330), both with a mesh size of 1 mm. Less frequently, a Monaco trawl (M) with a length of 6 m and a mesh size of 3 cm was used.

To collect fish eggs, larvae and other plankton, either open conical stramin nets (three different opening diameters; 100 cm, 150 cm and 200 cm – S 100, S 150 and S 200, respectively) with a mesh size of 1 mm, or fine-meshed open silk nets (P 100 and P 30) with mesh sizes of 333 µm and 76 µm were used. The silk nets were either used for surface sampling or they were attached to a trawl-wire used in greater depths.

Vertical plankton hauls were performed with Nansen closing nets (opening diameters of 30 cm (N 30) and 50 cm (N 50)) and a mesh size of 76 μm.

Four different types of ring trawls were used, with diameters from 130 cm to 1000 cm (C 200, C 130, E 300, E 1000 – the latter two only during the Dana expedition) and varying mesh sizes. The ring trawls were used for horizontal trawling, but could be closed at a specific depth before hauling them to the surface, thus allowing sampling at selective depths.

Bottom dredges comprised one with a rectangular opening (D 1), one with a triangular opening (D 2), and a small hand-dredge (H) with dimensions of 18 x 14 cm.

Additional types of sampling gear used occasionally during the "Thor" expedition included a small type of otter-trawl, a Danish eel hand-seine dragged by hand (mesh size of 1–2.5 cm) and shrimp nets used during wading from the beach for capturing various small fish, among others (Table 3).

The most commonly used gears during the expeditions were the young fish trawls, stramin nets, silk nets and Nansen nets (Table 4).

Several samples are not included in Table 4 for the following reasons:

- Wesenberg-Lund (1939) lists information on the sampling gear for each sample of pelagic polychaetes; however, in some cases it seems that samples from different gears were merged, as two different gears are listed per sample. These cannot be allocated unambigously.
- Fage (1920) lists sampling gears used to catch fish (Clupeiformes) in great detail when related to the "Thor" expeditions 1908–1910; however, for several samples proceeding from complementary expeditions the only information given is "Petersen's young fish trawl", without further specification. By matching samples to the information in the introductory volume, information on the opening diameter could be assigned to most samples. The remaining samples are listed in Table 4 as "Y".
- No information on the sampling gear used for the collection of calcareous algae is given by Lemoine (1915); however, by matching the samples to the information given in the introductory table (Schmidt 1912), information could be inferred for several of the samples. However, samples could not always be unambiguously matched, thus explaining the low numbers in Table 4.

*Environmental parameters*

During the "Thor" expedition, environmental conditions occurring at the time of the sampling were measured in great detail. Measurements are reported in parts of Volume 1 and Volume 3 of the series (Table 1). These were not digitized here, and the only environmental parameters contained in the dataset are those reported in the introductory table by Schmidt (1912). Additional information on the methodology was obtained from Nielsen (1912), same volume (Table 5).

### Quality control

Individual datasets were digitized manually from scanned copies of the original documents. In general, the quality control procedures applied through the data management process, were based on the Principles of Data Quality according to Chapman (2005)​ and the OBIS node manual. Moreover, as the digitization of historical datasets scattered over different manuscripts in a heterogeneous format presents a special challenge, more details on the digitization steps, the structure of the data and the quality control measures are given below (section "Step description").

### Step description

***Digitization steps***

1. Manual digitization of the information on samples and their associated metadata from the “Table of Stations” (page 25 and onwards) in the introductory volume (Schmidt 1912). These data were enriched with information from the main text (e.g. sampling methods and gear) when necessary.
2. Independent manual digitization of the three biological publications, including all available data on samples, sampling metadata, species occurences, abundances, etc.
3. Independent quality control of each dataset, e.g. check if location of coordinates falls on land or outside the study area; standardize taxon names against WoRMS (WoRMS Editorial Board 2016); check for inconsistencies in dates, depths, locations, sample numbers, abundances, use of gear, etc.
4. Sampling information in the biological publications (e.g. location, date, time, gear) were matched against the introductory volume which was used as a reference dataset, and unique sample IDs were ascribed to these samples. In cases where the biological publications included samples from additional expeditions which were not covered in the introductory volume, samples could not be matched (Fig. 3). There were also samples mentioned in the introductory volume which were not associated with any biological information, since they are described in other publications of the series that were not digitized yet.
5. A second round of quality control was performed on the integrated datasets. In cases of inconsistencies of information between the biological datasets and the introductory volume it was assumed that the information in the introductory table is the correct one, and a note was kept in the remarks field of the dataset.
6. Missing data in a dataset (on e.g. coordinates, sampling gear, sampling time, etc.) were either derived from other datasets or from other scientific and historical publications whenever possible.
7. Creation of metadata for each dataset.

This strategy resulted in a uniform dataset which allowed the seamless integration of data, as they all resulted from the same core expedition. However, to pay respect to the individual data publications and to allow users to access the data under a specific taxonomic focus, the datasets were published separately, with separate metadata and individual URLs. All datasets are available as Darwin Core Archives under a CC-Zero waiver via the Integrated Publishing Toolkit (IPT) of the Mediterranean Ocean Biogeographic Information System, supported by the LifeWatchGreece Infrastructure (see section "Data resources" below for the URLs of each dataset).

Data have to be formatted according to the DarwinCore schema (DwC) for dissemination through global biogeographic databases (e.g. Ocean Biogeographic Information System (OBIS), Global Biodiversity Information Facility (GBIF)), thus some modifications of the or enrichments were unavoidable. As this format is not necessarily user-friendly for human users (e.g. information might be merged into one field), a version of the datasets in a non-standardized but more easily usable format is provided as Suppl. materials 1, 2, 3, 4

***Difficulties and problems encountered during the procedure***

*Same station codes for different locations*

The samples of the core expedition of "Thor" in 1908–1910 had been given incrementing numbers in the introductory volume. However, the same identification codes had been used for stations of the 1905–1906 expedition, thus Schmidt created unique station codes by appending the last two digits of the year to these stations (e.g. "59.06" for station 59 in the year 1906). This practice was followed during the digitization for all years and stations from all publications where only the station code was listed. In cases where this was not sufficient to create a unique station ID, an additional letter was added to the station code (e.g. 283a.11 and 283b.11). This was done in two cases: (a) where the same code was used for different stations on different expeditions in the same year, and (b) where in the list of stations two different spatial points (coordinates) were listed under the same station code.

*Samples not identified unambiguously in the manuscript*

No unique sample codes were given in any of the publications. During the digitization process, unique codes were created for all samples (a sample was assumed to represent a unique combination of position, date, time, gear, length of wire and haul duration) by appending a letter to the station number (e.g. 10.08-a, 10.08-b, etc). In one case, where the same sample (all data identical) was listed twice in the introductory table, this was treated as two different samples to be in accordance with the manuscript; however, the biological data could not be mapped unambiguously to either of the two. During the assignment of sample IDs, records from the individual dataset were matched to the introductory table which served as a reference. This allowed to identify samples unambiguously and to supplement sampling metadata where it was missing in some cases. However, not all samples in the datasets “Pelagic Polychaetes” and “Calcareous algae” could be matched unambiguously to a sample code in the introductory table, even if they were taken at the same day and at the same station. In the polychaete dataset, many samples (gears/times) seem to have been merged (i.e. two sampling gears/times reported), or the sampling gear might have been matched to one sample and the sampling time to another. The dataset on calcareous algae did not report information on sampling time and gear. As the database requires a unique sample code, however, new “artificial” sample codes were created for these ambiguous samples. This probably increases the number of sampling events artificially, but as the total number of these “artificial” samples is low (20), this was considered as the best practice.

*Coordinates format and precision, georeferencing*

Latitude and Longitude are given in Degrees-Minutes in the original publications. This corresponds —roughly, depending on latitude— to a radius of uncertainty of around 2,000 m around the given point. In the datasets, coordinates were converted to decimal degrees with two decimals places to account for the originally given precision (no seconds given). The radius of uncertainty was recorded in the field "coordinateUncertaintyInMeters" in the DwC files. However, many of the samples were trawl catches and the ship was moving during the time of sampling over a distance of 1–2 nautical miles per hour [“… *towed after the ship for a shorter or longer time, usually ½ to 1 hour, the ship sailing about 2 knots in the hour*…”, in Schmidt (1912), p. 13]. These trawls were not represented in the data (only one point of coordinates was given, presumably the starting point). This additional uncertainty of the position resulting from the trawling has not been incorporated in the data, and the position of each sampling station must be considerd as an approximation.

In a few cases, no coordinates but a named location was given. These locations were georeferenced, providing coordinates for latitude and longitude, as well as a radius of uncertainty (“coordinateUncertaintyInMeters”), using the Edit Point location tool.

Several reported coordinates fell on land. Some of those represented errors in the manuscript that could be resolved from the context (e.g. East and West were interchanged) and these were corrected (original coordinates reported in the remarks field), but some stations still fell on land and their correct position could not be determined. All the subsequent publications have used the same, obviously false, coordinates, and the included maps often do not have a sufficient precision to georeference the stations correctly.

In addition, some stations were listed with the same coordinates in the introductory table but were actually placed at different positions according to the map in the introductory volume. As it could not be determined whether the map or the table were correct, and the map does not allow georeferencing with a great accuracy, the coordinates were kept as they were listed in the original table with a remark.

*Time format*

The publications were not consistent in their use of “am” and “pm” when noon/midnight were concerned. Times could be given as 0 pm or 12 pm for noon and 12 am or 0 am for midnight, but not always in the same manner. The correct conversion into the 24 h format could however be determined from the sequence of samples.

*Depths*

Although depths were given for each station, it was not always clear whether these were bottom depths or sampling depths. In most cases, they appeared to be bottom depths, while in other cases they were listed for each sample and differed per station, or they were clearly sampling depths (e.g. 0–1 m at a deep water station). Data exchange schemes such as Darwin Core only provide fields for sampling depths. As a compromise, and to avoid false conclusions (i.e. interpreting bottom depth as sampling depth), sampling depths were filled in only when unambiguous, using complementary information from the depth column, the gear (e.g. benthic, pelagic) and the length of the wire used. All other depths were included in the remarks field of each sample, leaving their interpretation to the users of the data.

Often, depth ranges, or either minimum or maximum depths only (e.g. >3,000 m) were recorded. This may have resulted from difficulties associated with precise depth measurements during that era: although depth could be accurately determined by the sounding of a lead ball, the interference of other conditions such as weather, currents, the available wire length and the nature of the bottom often might have allowed only for estimates, ranges or minimum values. In addition, multiple depth values per station may have resulted from the working practice on board (see also Schmidt 1912 for a description of a typical working day on board). Coordinates were usually recorded once, before sampling started. During subsequent measurements, the boat moved away from its initial position, either actively during trawling, or passively (drifting). Any subsequent depth measurement in the area was still considered to belong to the same station, but was effectively performed at a different spatial location. Geographically, any location on earth has only one depth or altitude, thus when translating the information from the manuscript into a data schema, information loss occurred, as only one bottom depth can be associated with one position.

*Environmental information*

Data for the "nature of bottom", air temperature and chlorine concentration were missing for stations after ID 297.11 in the middle of an expedition (page 48), and since no explanation was provided in the manuscript this is suspected to be a typesetting error. In some cases, environmental parameters were reported but their measurement protocols were unknown (Table 5), or the data were derived from subjective estimates (e.g. weather and sea state reports); nevertheless, all this information was made available even with limitations and/or bias, considering this would offer an added value to the completeness of the dataset.

*Ditto marks and abbreviations*

**Figure 8.**
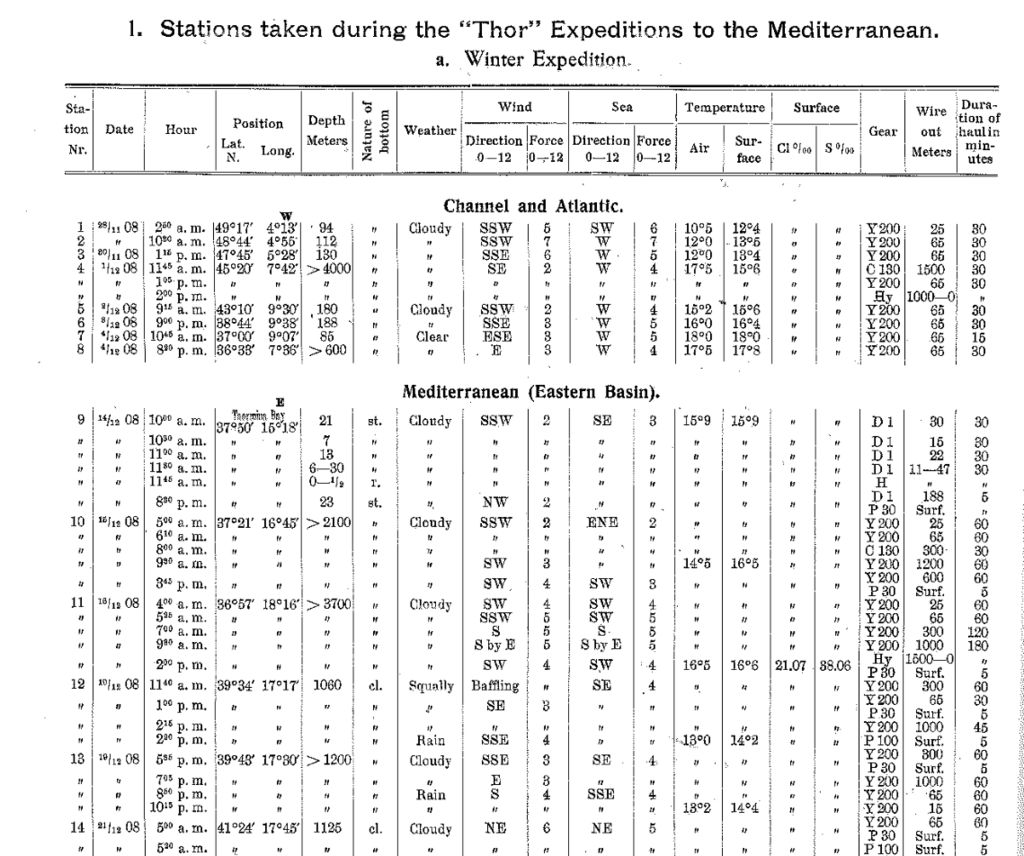
Excerpt from the list of samples of the "Thor" Expedition (taken from Schmidt 1912), showing the arrangement of information ​and the inconsistent use of ditto marks.

Many table cells of the original manuscript contained ditto marks (") (Fig. 8), a typesetting symbol meaning "same value as above". In the "Thor" publications this made sense in many cases (e.g. for the station number, date or coordinates), but in other cases the ditto marks seemed to represent "no data" (e.g. when the first rows of the table contain ditto marks for environmental measurements without any previous value being present). Sometimes (e.g. for gear, duration of haul, and depth), it was difficult to understand whether the symbol represented "same as above" or "no data". During the data digitization process the ditto marks were always treated as "same as above" for the station number, date, coordinates, nature of the bottom, time, depth, sampling gear, duration of haul and wire length, and they were replaced by the values of the preceding record in the introductory table (when present). However, for weather, wind force and direction, sea force and direction and environmental parameters, the ditto marks were interpreted as "no data". In all cases where there seemed to be errors in the original manuscript regarding the use of ditto marks (e.g. some depths were impossible or did not make sense in the context) a remarks field was included in the dataset, leaving the interpretation of the data to the user. Ditto marks were also present in the paper on Engraulidae and Clupeidae, but there a distinction was made in the use of the symbols: "no data" was signified by two dots (..), and "see above" by ditto marks (").

*Size classes*

The paper of Fage (1920) on Clupeiformes contained larval size classes. These were reported as distinct records in the dataset. The size class was added in the field “lifeStage". Size classes were consistent within some species (e.g. *Sardina pilchardus* [as *Clupea pilchardus* in publication]), but not within others (e.g. *Sprattus sprattus* [as *Clupea sprattus* in publication]), neither between species. Size classes used for sprat larvae were different from size classes for other species.

*Inconsistencies in the introductory table*

In the samples collected from the Strait of Messina in 1911 (page 47), depths were reported in brackets, and often as ranges (e.g. “10–60”). Although the meaning of this notation remains unclear, presumably these were an estimation of sampling depths, as bathymetric maps show these sampling areas to be much deeper than 60 m. In many of these samples, station codes do not correspond to coordinates: one station codes may have several different coordinates, or the same coordinates were sometimes given to different station codes. During digitization, new station codes were assigned following the strategy described above (paragraph "Same station codes for different locations").

The number of rows per column in the introductory table was not always consistent; containing, for instance, three different depths and sampling times and four different sampling gears. In these cases, it was often unclear which information belonged to which sampling event. Usually, the additional information row was considered to belong to the previous one. Although this strategy may have introduced wrong information into the dataset there was no other possible solution for such issue. Similar issues were also encountered in some of the biological publications.

*Data inconsistencies within and across datasets*

The structure of the publications is prone to create inconsistencies in the data, both across publications and within publications. Data on the same samples were published across several volumes over many years. Each publication repeated all or parts of the sampling information mentioned in the introductory table, but often with small differences (e.g. slightly different sampling times, depths, gears) or even with typographic errors. As it is unknown from where individual authors received this metadata on sampling —from the introduction of Schmidt (1912) which all subsequent authors consulted as a reference, or from handwritten notes which were re-transcribed and published over and over again— the correct information for a sample cannot be determined. Similarly, inconsistencies occurred also within some datasets: as occurrences are typically grouped by taxon, metadata on a sample (e.g. date, time, depth, sampling gear) were repeated over and over again for many taxa, thus introducing errors and inconsistencies. This was particularly pronounced in the dataset “Pelagic polychaetes”. During the digitization of these datasets, the introductory table has been treated as a “master table” and was assumed to be correct, and all other samples were matched to this reference.

## Geographic coverage

### Description

Coordinates given here are for the four datasets combined. For information on the coverage of individual datasets see Table 2

### Coordinates

0.52 and 59.32 Latitude; 33.05 and -29.88 Longitude.

## Taxonomic coverage

### Description

**Figure 9.**
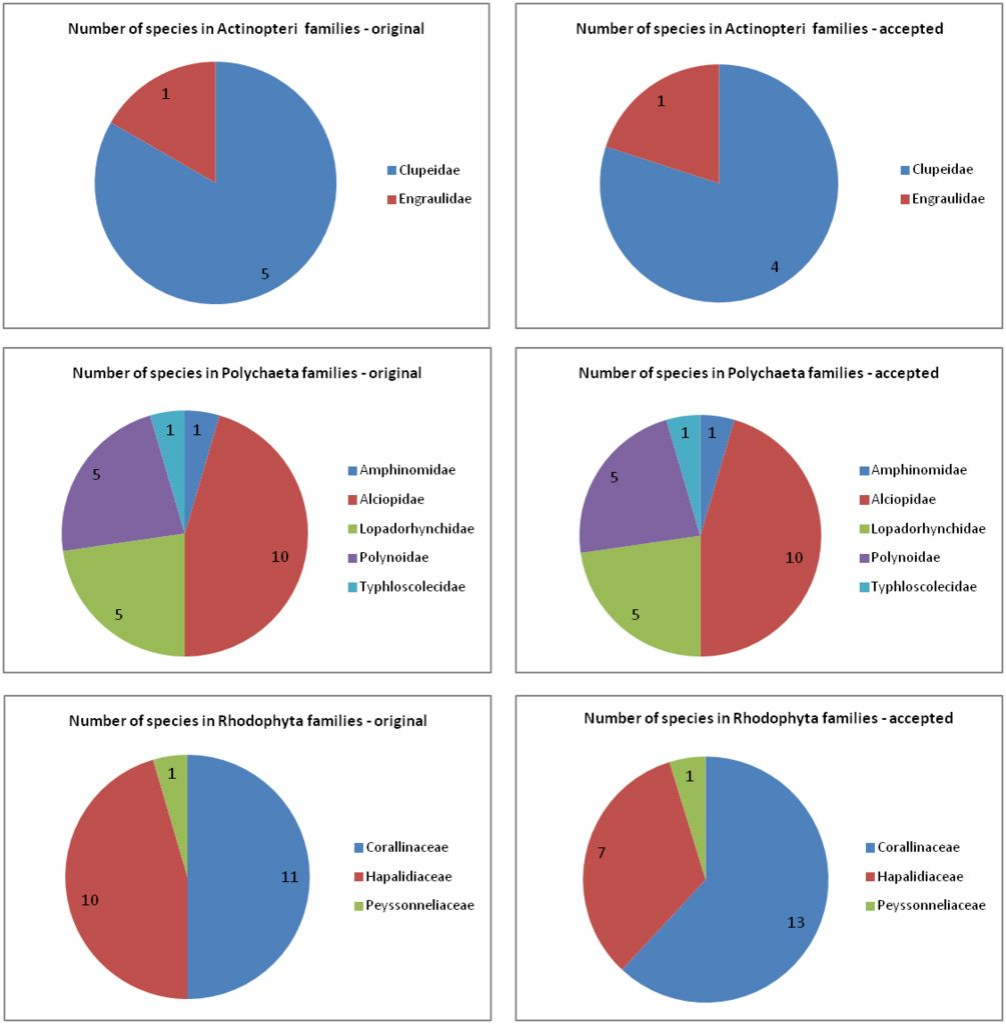
Number of species for Actinopteri, Polychaeta and Rhodophyta groups according to the originally published (left) and the currently accepted (right) classification systems.

The datasets examined in this paper include two families of Actinopteri, five families of Polychaeta and three families of Rhodophyta (Fig. 9). Differences were observed between the taxonomic names of the species under which they were reported in the original biological publications and in their currently accepted status according to WoRMS.

In the Polychaeta, several taxa are nowadays reported under different names, but the number of taxa per family reported remains stable: 10 species belonged to the family Alciopidae, 5 to the Polynoidae, 5 to the Lopadorrhynchidae, 1 to the Amphinomidae and 1 to the Typhloscolecidae.

In the Actinopteri, 4 species belong to the Clupeidae and 1 to the Engraulidae. Records for *Clupea sprattus* and *C. sulinae* were merged under the name *Sprattus sprattus*, according to their currently accepted taxonomy. Taxa not identified to species level were reported under the lowest possible rank (e.g. Clupeiformes).

The most significant reclassifications were observed in the Rhodophyta where only 6 species (out of 21) had still the same scientific name and were members of the same family. The 11 species that were originally part of the Corallinaceae family in Rhodophyta have increased to 13, while the 10 species of Hapalidiaceae have decreased to 7 species. Three species that were members of the Hapalidiaceae family belong now in the Corallinaceae family, while 6 species that used to be part of Corallinaceae are now in Hapalidiaceae.

### Taxa included

**Table:** 

Rank	Scientific Name	
class	Polychaeta	
order	Clupeiformes	
phylum	Rhodophyta	

## Temporal coverage

**Data range:** 1904 9 05 – 1930 6 18.

### Notes

This time range covers the samples of all four datasets digitized here. For details on individual datasets see Table 2 .

## Usage rights

### Use license

Creative Commons Public Domain Waiver (CC-Zero)

## Data resources

### Data package title

Report on the Danish Oceanographical Expeditions 1908-1910 to the Mediterranean and adjacent seas

### Resource link

http://ipt.medobis.eu/resource?r=thorexpeditionintroduction

### Number of data sets

4

### Data set 1.

#### Data set name

Introduction. Report on the Danish Oceanographical Expeditions 1908-1910 to the Mediterranean and adjacent seas

#### Data format

Darwin Core Archive

#### Number of columns

30

#### Character set

UTF-8

#### Download URL

http://ipt.medobis.eu/resource?r=thorexpeditionintroduction

#### Description

This dataset comprises the location of sampling stations, sampling information (methods) and some environmental data recorded during the Danish expeditions to the Mediterranean and adjacent seas 1908-1910. The data were digitized from the report of Schmidt (1912). This table was created and used as reference for the biogeographic data, which were published in three multi-part volumes. Three of these volumes with biogeographic data have been digitized already and are available through the MedOBIS IPT installation.The Darwin Core Archive comprises two files with data: *event.txt* and *measurementorfact.txt*. The measurementorfact.txt file is linked to the event.txt file through the column "id".

**Data set 1.** 

Column label	Column description
id	Internal database ID. In event.txt identical to eventsID and constituting a unique identifier for the event (= sampling event). In measurementorfact.txt it is the ID of the event at which the measurement was taken.
eventID	Unique ID for the record, identical to id.
parentEventID	An identifier for the broader event that groups this and potentially other events – in this case, a link to the database internal ID of the station and included in the file only for compliance with the schema.
samplingProtocol	A free-text description of the method/ protocol used during the sampling event.
sampleSizeValue	A numeric value for a measurement of the size (time duration, length, area, or volume) of a sample in a sampling event. Does not contain values here, included only for compliance with the schema.
sampleSizeUnit	The unit of measurement of the size (time duration, length, area, or volume) of a sample in a sampling event. Does not contain values here, included only for compliance with the schema.
samplingEffort	Sampling time in minutes
eventDate	The date and time of sampling, recorded in standard format (ISO 8601:2004).
year	The year of sampling (four digits).
month	The month of sampling (one or two digits).
day	The day of the month of sampling (one or two digits).
habitat	A description of the habitat of the sample, in this case representing the information contained in the column "Nature of Bottom" of the original publication.
fieldNumber	A unique sampling code (across the digitized datasets). Here composed of the locationID and year and an incrementing small letter to distinguish between different sampling events at the same station in the same year.
eventRemarks	The name of research vessel, the bottom or sampling depth given and other remarks on the original text. Includes also any information from the original publications which was corrected during the digitization in cases it was clearly incorrect.
locationID	The station code as reported on the original text plus the sampling year, in some cases with slight modifications in order to avoid the same station code being used for different locations.
locality	The name of the locality of the sampling station, if given in the publication.
minimumDepthInMeters	The minimum (shallowest) sampling depth of the event.
maximumDepthInMeters	The maximum (deepest) sampling depth of the event.
locationRemarks	Comments or notes about the location and its coordinates.
decimalLatitude	The geographic latitude (in decimal degrees, using the spatial reference system given in geodeticDatum) of the geographic center of a Location. Positive values are north of the Equator, negative values are south of it.
decimalLongitude	The geographic longitude (in decimal degrees, using the spatial reference system given in geodeticDatum) of the geographic center of a Location. Positive values are east of the Greenwich Meridian, negative values are west of it.
coordinateUncertaintyInMeters	A radius of uncertainty around the given coordinates (in metres). The true location / coordinates may fall anywhere within that circle.
measurementID	A unique identifier for the MeasurementOrFact (information pertaining to measurements, facts, characteristics, or assertions).
measurementType	The type of the measurement (e.g. Chlorine at the water surface)
measurementValue	The value of the measurement.
measurementAccuracy	he description of the potential error associated with the measurementValue
measurementUnit	The unit associated with the measurementValue.
measurementDeterminedBy	Person(s) who determined the measurementValue.
measurementMethod	A description of or reference to (publication, URI) the method or protocol used to determine the measurement
measurementRemarks	Comments or notes about the measurement.

### Data set 2.

#### Data set name

Danish Oceanographical expeditions 1908-1910 to the Mediterranean and adjacent seas-Pelagic Polychaetes

#### Data format

Darwin Core Archive

#### Number of columns

25

#### Character set

UTF-8

#### Download URL

http://ipt.medobis.eu/resource?r=thorexpedition_pelagicpolychaetes

#### Description

The present historical paper deals with the pelagic Polychaetes except the Tomopterids collected on the cruises of the "Thor", 1908-1910 in the Mediterranenan and adjacent waters. The tables included in this report present also the scientific results from other research vessels such as "Dana" (years 1921 and 1930) and "S/S Pangan" (1911).

The Darwin Core Archive comprises two files with data: *event.txt* and *occurrence.txt*. The occurrence.txt file is linked to the events.txt file through the column "id". The data fields in event.txt, as well as some in the file occurrence.txt are the same as in the dataset "Introduction. Report on the Danish Oceanographical Expeditions 1908-1910 to the Mediterranean and adjacent seas". Any fields already described above in the section for that dataset are not repeated here.

**Data set 2.** 

Column label	Column description
id	An identifier for the sampling event, linking to the column "id" in the event.txt file.
institutionCode	The name (or acronym) of the institution providing and curating the record.
datasetName	The name of the data set from which the record was derived
basisOfRecord	Required by the DarwinCore schema, describing the origin of the data record. Here, all are "Human Observation".
occurrenceID	A unique identifier (within the dataset) for the occurrence record.
catalogNumber	A constructed (globally) unique identifier, combination of dataset name and fieldNumber.
occurrenceRemarks	Any remarks, notes, comments on the occurrence.
individualCount	The number of individuals of the taxon in the sample.
sex	The biological sex of the taxon.
lifeStage	The life stage of the taxon.
preparations	Preparations of the sample (e.g. preservation in ethanol). Empty here, included for completeness of the schema.
identifiedBy	The person(s) who identified the taxon.
identificationReferences	Bibliographical references used for the identification. Empty here, included for completeness of the schema.
scientificNameID	A unique identifier for the name of the taxon, here the LSID of the World Register of Marine Species.
scientificName	The full scientific name retrieved from the World Register of Marine Species, after matching the taxon name as in the original publication. Contains the value of the field "ScientificName" as retrieved after using the function "Match taxa" of WoRMS.
kingdom	The name of the kingdom in which the taxon is classified. If the taxon was not found in WoRMS at the time of taxon matching, the field is empty.
phylum	The name of the phylum in which the taxon is classified. If the taxon was not found in WoRMS at the time of taxon matching, the field is empty.
class	The name of the class in which the taxon is classified. If the taxon was not found in WoRMS at the time of taxon matching, the field is empty.
order	The name of the order in which the taxon is classified. If the taxon was not found in WoRMS at the time of taxon matching, the field is empty.
family	The name of the family in which the taxon is classified. If the taxon was not found in WoRMS at the time of taxon matching, the field is empty.
genus	The name of the genus in which the taxon is classified. If the taxon was not found in WoRMS at the time of taxon matching, the field is empty.
subgenus	The name of the subgenus in which the taxon is classified (if present).
specificEpithet	The specific epithet of the taxon (if at species level).
nomenclaturalCode	The nomenclatural code under which the scientificName is constructed. Included here only for compliance with the schema.
taxonomicRemarks	Any remarks on the taxon, contains also the exact version of the taxon as written in the original publication.

### Data set 3.

#### Data set name

Engraulidae-Clupeidae. Report on the Danish Oceanographical Expeditions 1908-1910 to the Mediterranean and adjacent seas

#### Data format

Darwin Core Archive

#### Number of columns

1

#### Character set

UTF-8

#### Download URL

http://ipt.medobis.eu/resource?r=thorexpedition_engraulidaeclupeidae

#### Description

Larvae of fish from families Engraulidae and Clupeidae were sampled. Sampling was mainly pelagig, thus mainly by Petersen's young fish trawl. Data proceed from the Thor expeditions 1908-1910 but data from other expeditions (Thor 1905 and 1906) and other vessels (Nordboen and Pangan) are incorporated in the report, encompassing years 1904-1911

The Darwin Core Archive comprises two files with data: *event.txt* and *occurrence.txt*. The occurrence.txt file is linked to the events.txt file through the column "id". The data fields in event.txt, as well as some in the file occurrence.txt are the same as in the dataset "Introduction. Report on the Danish Oceanographical Expeditions 1908-1910 to the Mediterranean and adjacent seas". The file occurrence.txt contains exactly the same fields as the file occurrence.txt in the dataset "Danish Oceanographical expeditions 1908-1910 to the Mediterranean and adjacent seas-Pelagic Polychaetes". Thus, the description of all fields are not repeated here.

**Data set 3.** 

Column label	Column description
all columns	All columns have already been described above in the datasets "Introduction. Report on the Danish Oceanographical Expeditions 1908-1910 to the Mediterranean and adjacent seas" and "Danish Oceanographical expeditions 1908-1910 to the Mediterranean and adjacent seas-Pelagic Polychaetes" and are not repeated here.

### Data set 4.

#### Data set name

Calcareous Algae. Report on the Danish Oceanographical Expeditions 1908-10 to the Mediterranean and adjacent seas

#### Data format

Darwin Core Archive

#### Number of columns

1

#### Character set

UTF-8

#### Download URL

http://ipt.medobis.eu/resource?r=thorexpedition_k1_calcareous_algae

#### Description

This dataset is based on the paper entitled “Calcareous Algae. Report on the Danish Oceanographical Expeditions 1908-10 to the Mediterranean and adjacent seas” published by Lemoine (1915). It comprises of 59 records of Rhodophyta (Corallinales and Peyssonneliales) collected during the 1908-9 and 1910 expeditions of Thor vessel from a wide geographical (from the Aegean Sea to the eastern Atlantic Ocean) and bathymetric range (3-98 m).

The Darwin Core Archive comprises two files with data: *event.txt* and *occurrence.txt*. The occurrence.txt file is linked to the events.txt file through the column "id". The data fields in event.txt, as well as some in the file occurrence.txt are the same as in the dataset "Introduction. Report on the Danish Oceanographical Expeditions 1908-1910 to the Mediterranean and adjacent seas". The file occurrence.txt contains exactly the same fields as the file occurrence.txt in the dataset "Danish Oceanographical expeditions 1908-1910 to the Mediterranean and adjacent seas-Pelagic Polychaetes". Thus, the description of all fields are not repeated here.

**Data set 4.** 

Column label	Column description
all columns	All columns have already been described above in the datasets "Introduction. Report on the Danish Oceanographical Expeditions 1908-1910 to the Mediterranean and adjacent seas" and "Danish Oceanographical expeditions 1908-1910 to the Mediterranean and adjacent seas-Pelagic Polychaetes" and are not repeated here.

## Additional information

**Conclusions**

This work represents a first step towards the digitization and harmonization of a complex set of related information distributed across different historical publications. Making legacy data publicly available is an increasing need (and duty of scientists), with expected effects on the scientific and societal perception of biogeography. The conclusions provided here can offer lessons learnt through this experience, but they can also contribute to any future attempt for rescuing legacy data and they can provide a sound basis for the digitization of the complete set of publications derived from the Danish expeditions (Table 1).

When legacy data are concerned, their integration into a harmonized, comparable format and their quality control is a special challenge. Data might be inconsistent across different publications, or even within the same publication, and obvious errors are identified during digitization. Often, information is missing, or spread across several publications. The meaning of symbols may be unclear, even when they seem to have an international standard nowadays (e.g. the meaning of ditto marks). Sometimes, these problems can be solved by tracking down the information in other publications, or by logically deducing information. This requires a sound knowledge of sampling techniques and characteristics of the sampled area and habitat. However, as the original authors cannot be consulted anymore, some of these problems will remain unresolved, and currently there is no standard strategy or “best practice” on how to deal with such issues.

During the data harmonization and quality control of the data it was required to take decisions related to species names (reported vs currently accepted), names of water basins (previous vs recent definitions), georeferencing (precision related to the transformation of coordinates), and to check additional documents for eventual integration of data. An important lesson learnt in this respect was the need of a team to integrate knowledge and perspectives, and the involvement of experts of different disciplines, able to interpret data correctly and reduce errors in the final dataset.

***Outlook***

Biodiversity legacy literature contains a wealth of information on the biosphere and can provide valuable insights into the past state of the world's ecosystems. However, the extraction of this information is tedious and time-consuming and requires skilled personnel (Faulwetter et al. 2016). Multi-disciplinary working groups should therefore be formed to develop new methods to harvest and use this information: librarians and natural history museums may initiate and facilitate access to such information and publications, data scientists to informaticians are needed to develop tools and workflows to semi-automate the extraction of information, and biologists, modellers and even social scientists should collaborate to harness this information in global models, giving insights into global change and their consequences for society.

## Supplementary Material

Supplementary material 1 Thor - "Introductory table" Data type: sampling metadata and environmental information
Brief description: As the Darwin Core version of this dataset is rather complex in structure, this is a more user-friendly version of the introductory table of the "Thor" expedition, digitized from Schmidt (1912), in csv format (tab-delimited).
File: oo_109397.csv Sarah Faulwetter, Marilena Tsompanou

Supplementary material 2 Thor - "Pelagic polychaetes" Data type: occurrence data
Brief description: As the Darwin Core version of this dataset is rather complex in structure, this is a more user-friendly version of the occurrence records of the pelagic polychaetes of the "Thor" and other expeditions, digitized from Wesenberg-Lund (1939), in csv format (tab-delimited).
File: oo_109400.csv Sarah Faulwetter, Dimitra Mavraki

Supplementary material 3 Thor - "Clupeiformes" Data type: occurrence data
Brief description: As the Darwin Core version of this dataset is rather complex in structure, this is a more user-friendly version of the occurrence records of the Clupeiformes of the "Thor" and other expeditions, digitized from Fages (1920), in csv format (tab-delimited).
File: oo_109401.csv Sarah Faulwetter, Lucia Fanini

Supplementary material 4 Thor - "Rhodophytes" Data type: occurrence data
Brief description: As the Darwin Core version of this dataset is rather complex in structure, this is a more user-friendly version of the occurrence records of the rhodophytes of the "Thor" expedition, digitized from Lemoine (1915), in csv format (tab-delimited).
File: oo_109402.csv Sarah Faulwetter, Vasilis Gerovasiliou

Supplementary material 5 Data from Fishermen of Colliure Data type: fisheries landings data
Brief description: These data are included in Fage's (1920) report on the Clupeiformes of the Thor expedition. Fage reports (page 63) the sardine catch of the “Fishermen of Colliure”, containing informal and unpublished information related to the timing of the capture of the sardine (years 1902-1913), to be compared with the direct data gathered from the expedition. The data are related to species, mentioned with the vernacular name of “sardine”, with biomass data (Kg) on a monthly base, covering 12 years (1902-1913) of fishery. As the coordinates of the specific catching area were not reported, the location of Colliure (https://en.wikipedia.org/wiki/Collioure) was used as geographical reference.
Empty cells mean “no information in the text”. It may mean no captures, or no fishing. Data are in csv format (tab separated).
File: oo_109403.csv Lucia Fanini

## Figures and Tables

**Table 1.** List of all papers published in the series "Report on the Danish Oceanographical expeditions 1908-1910 to the Mediterranean and adjacent seas". For Volume 2, parts B, F and G, no reports were published.

**Volume**	**Part**	**Title**	**Author**	**Year**
V1	1	Introduction, hydrography, deposits of the sea-bottom	Johannes Schmidt	1912
V2 Biology	A1	Flat-fishes (Heterosomata)	Harry Mcdonald Kyle	1913
V2 Biology	A2	Sternoptychidae (*Argyropelecus* and *Sternoptyx*)	Poul Jespersen	1915
V2 Biology	A3	Shore-fishes	Louis Fage	1918
V2 Biology	A4	Stomiatidae (*Stomias*)	Vilhelm Ege	1918
V2 Biology	A5	Argentinidae, Microstomidae, Opisthoproctidae, Mediterranean Odontostomidae	Johannes Schmidt	1918
V2 Biology	A6	Mediterranean Bramidae and Trichiuridae	Johannes Schmidt and A. Strubberg	1918
V2 Biology	A7	Mediterranean Scopelidae (*Saurus*, *Aulopus*, *Chlorophthalmus* and *Myctophum*)	Åge Vedel Tåning	1918
V2 Biology	A8	* Lepadogaster *	Frédéric Guitel	1920
V2 Biology	A9	Engraulidae, Clupeidae	Louis Fage	1920
V2 Biology	A10	* Lophius *	Åge Vedel Tåning	1923
V2 Biology	A11	Scombriformes	Ernst Ehrenbaum	1924
V2 Biology	A12	Mediterranean Sternoptychidae	Poul Jespersen and Åge Vedel Tåning	1926
V2 Biology	A13	Sudidae (*Paralepis*)	Vilhelm Ege	1930
V2 Biology	A14	Carangidae	W. Schnakenbeck	1931
V2 Biology	C1	Cephalopoda	Eduard Degner	1926
V2 Biology	D1	Isopoda, Tanaidacea, Cumacea, Amphipoda (excl. Hyperiidea)	Knud Stephensen	1915
V2 Biology	D2	Hyperiidea-Amphipoda (pt. 1: Lanceolidae, Scinidae, Vibiliidae, Thaumatopsidae)	Knud Stephensen	1918
V2 Biology	D3	Decapoda-Macrura excl. Sergestidae (Penaeidae, Pasiphaeidae, Hoplophoridae, Nematocarcinidae, Scyllaridae, Eryonidae, Nephropsidae, appendix)	Knud Stephensen	1923
V2 Biology	D4	Hyperiidea-Amphipoda (pt. 2: Paraphronimidae, Hyperiidae, Dairellidae, Phronimidae, Anchylomeridae)	Knud Stephensen	1924
V2 Biology	D5	Hyperiidea-Amphipoda (pt. 3: Lycaeopsidae, Pronoidae, Lycaeidae, Brachyscelidae, Oxycephalidae, Parascelidae, Platyscelidae)	Knud Stephensen	1926
V2 Biology	D6	Euphausiacea	Johan T. Ruud	1936
V2 Biology	E1	Pelagic polychaetes of the families, Aphroditidae, Phyllodocidae, Typhloscolecidae and Alcioidae.	Elise Wesenberg-Lund	1939
V2 Biology	H1	Medusae	Paul Lassenius Kramp	1924
V2 Biology	H2	Siphonophorae	H. B. Bigelow and M Sears	1937
V2 Biology	J1	Mediterranean Ceratia	Eugen Jörgensen	1920
V2 Biology	J2	Mediterranean Dinophysiaceae	Eugen Jörgensen	1923
V2 Biology	J3	Mediterranean Tintinnidae	Eugen Jörgensen	1924
V2 Biology	J4	Bacillariales	J. Pavillard	1926
V2 Biology	K1	Calcareous algae	Mme Paul Lemoine	1915
V2 Biology	K2	Sea-grasses	C. H. Ostenfeld	1918
V2 Biology	K3	Algae (excl. calcareous Algae)	Henning E. Petersen	1918
V3 Miscellaneous papers	1	Experiments with drift-bottles : first report	Johannes Schmidt	1913
V3 Miscellaneous papers	2	The Sargasso Sea, its boundaries and vegetation	Øjvind Winge	1923
V3 Miscellaneous papers	3	On the quantity of macroplankton in the Mediterranean and the Atlantic	Poul Jespersen	1923
V3 Miscellaneous papers	4	Elvers from north and south Europe	A.C. Strubberg	1923
V3 Miscellaneous papers	5	Experiments with drift-bottles (second report)	Giovanni Platania	1923
V3 Miscellaneous papers	6	Nitrate and phosphate contents of the Mediterranean water	H. Thomsen	1931
V3 Miscellaneous papers	7	Some quantitative investigations on the bottom fauna at the west coast of Italy, in the Bay of Algiers, and at the coast of Portugal.	Ragnar Spärck	1931

**Table 2.** Summary of the content and coverage of the four digitized datasets. Note that depths may represent either sampling depths or bottom depths, this is unclear and confused in the publication (see also section "Step description" – "Difficulties and problems encountered during the digitization procedure")

	**Introductory table**	**Polychaetes**	**Fish (Clupeiformes)**	**Calcareous algae**
Temporal coverage	1905-06-13 to1912-01-07	1905-05-14 to1930-06-18	1904-09-05 to1914-01-24	1908-12-14 to1910-09-19
Geographical coverage (min, max Latitude / min, max Longitude)	30.33, 53.1 / -29.88, 29.83	0.516, 51.57 / -17.983, 28.23	28.6, 59.32 / -10.7, 33.05	30.38, 48.72 / -4.75, 26.1
Minimum depth	0	20	0	3
Maximum depth	6020	>4000	> 3700	98
No. of stations	443	210	208	16
No. of samples	1566	599	341	16
No. of occurrence records	–	883	646	59
Vessels	Thor, Ingolf, Florida, Pangan, St. Croix, St. Jan, St. Thomas, Agent Petersen, Anne, Caroline Kock	Thor, Pangan, Dana	Thor, Algarvae, Nordboen, Pangan	Thor

**Table 3.** Sampling gears used during the "Thor" (and complementary) expeditions. Abbreviations are used in the table of samples in Schmidt's introductory volume (Schmidt 1912) and are also explained therein. Additional information on mesh sizes of nets was retrieved from Sverdrup et al. (1942).

**Abbreviation**	**Full description**
Aa 2	Eel drift-seine
C 130	Ring-trawl, 130 cm in diameter at opening, 1 mm mesh size, horizontal haul
C 200	Ring-trawl, 200 cm in diameter at opening, 1 mm mesh size, horizontal haul
D 1	Dredge, rectangular opening, 27x117 cm, 1 mm mesh size, horizontal haul
D 2	Dredge, triangular opening, 45x45 cm, 1 mm mesh size, horizontal haul
H	Hand-Dredge, 18x14 cm
L	Long-line with halibut and cod hooks
M	Monaco trawl, 56x170 cm at opening, horizontal haul
N 30	Nansen's closing net, 30 cm in diameter at opening, gauze No. 20, 0.076 mm mesh size, vertical haul
N 50	Nansen's closing net, 50 cm in diameter at opening, gauze No. 20, 0.076 mm mesh size, vertical haul
O	Otter-trawl, head-rope 15.25 m (50 feet), 30 mm mesh size, horizontal haul
P 100	Silk-net, open, conical, 100 cm in diameter at opening, gauze No. 3, 0.333 mm mesh size
P 30	Silk-net, open, conical, 30 cm in diameter at opening, gauze No. 20, 0.076 mm mesh size
R	Shrimp net
S 100	Stramin-net, open, conical, 100 cm in diameter at opening, 1 mm mesh size
S 150	Stramin-net, open, conical, 150 cm in diameter at opening, 1 mm mesh size
S 200	Stramin-net, open, conical, 200 cm in diameter at opening, 1 mm mesh size
T 25	Taffeta-net, open, conical, 25 cm in diameter
Y 200	Young-fish trawl, 200 cm in diameter at opening, 1 mm mesh size, horizontal haul
Y 330	Young-fish trawl, 330 cm in diameter at opening, 1 mm mesh size, horizontal haul
E 1000	Open Ringtrawl, 1000 cm in diameter
E 300	Open Ringtrawl, 300 cm in diameter, meshes 24-18-12 mm
Y	Petersen trawl, without opening given

**Table 4.** Numbers of samples taken with each sampling gear during the "Thor" (and complementary) expeditions, per dataset. Samples where the gear used could not be determined (e.g. unknown abbreviation used, mixed gears reported per sample (samples merged), etc. – see also next paragraph) are excluded. Abbreviations are explained in Table 3.

**Abbreviation**	**Introductory table**	**Polychaetes**	** Clupeiformes **	** Calcareous Algae **
Aa 2	11			
C 130	3	1		
C 200	11	1	1	
D 1	65	1		6
D 2	6			1
E 1000		2		
E 300		2		
H	10			
O	1			
P 100	229	10	11	
P 30	87			
R	1			
S 100	76	6	9	
S 150	19	15	3	
S 200	27	345	13	
T 25	4			
Y		1	64	
Y 200	481	187	204	
Y 330	219	20	26	

**Table 5.** Environmental parameters reported for the samples in the introductory table by Schmidt (1912).

**Column name in publication**	**Units as reported in publication**	**Notes**
Weather		General meteorological conditions (cloudy, misty, clear etc.) are reported, but seem to be subjective, no scale of reference is reported anywhere.
Wind Direction0–12	Indicated in the column header as a 0–12 scale, but quadrants are reported (e.g. NNE; S)	0–12 is an unusual scale (commonly, degrees are used) and not used in the actual data. No further information available, maybe simply a typographic error in the column name.
Wind Force 0–12		Appears to be on a Beaufort scale, even if not specified in the original document.
Sea Direction0–12	Indicated to be on a 0–12 scale, but quadrants are reported (e.g. NNE; S).	Seems to be the observed direction of the swell, no further information given in the manuscript.
Sea Force0–12		Appears to be on a Beaufort scale, even if not specified in the manuscript(s) (The Douglas scale would have only 10 possible values).
Temperature Air	°C	Precision of one decimal place. Measurement device was not specified.
Temperature Surface	°C	Precision of two decimal places; given in the form of "12°40" in the manuscript.
Surface Chlorine	‰	Chlorine weight in grams per 1,000 g sea-water, unit specification from Nielsen (1912).
Surface Salinity	‰	Obtained by titration in the Mediterranean Sea and by aerometer in Bosphorus and the Dardanelles. Salinity weight in grams per 1,000 g sea-water Nielsen (1912).
